# Supplementary material for: Improving visualization of the cervix during pelvic exams: A simulation using a physical model of the speculum and human vagina as a steppingstone to reducing disparities in gynecological cancers
Source: PLoS One. 2023 Sep 26;18(9):e0283145. doi: 10.1371/journal.pone.0283145 (PMC10522035; doi:10.1371/journal.pone.0283145)
Supplement: S1 Table — (DOCX) [file pone.0283145.s002.docx]

S1 Table. Materials, software, and tools used in our experiments

| Physical materials | | |
| --- | --- | --- |
| Brief Name | Description | Full product name and manufacturer (if available) |
| Speculum | Clear, plastic (acrylic), bivalve, disposable, self-retaining, clean non-sterile, speculum, white yoke (see Figure 1A) | KleenSpec® Vaginal Specula  UPC #732094143690, VG-2501  Welch Allyn, Inc, Skaneateles Falls, New York, USA  Hill-Rom Holdings, Inc., Chicago, Illinois, USA |
| Camera | 12.2-megapixel rear camera of Android smartphone, resolution 4032px by 3024px, focal aperture f/1.5-2.4, focal length 26mm (wide), sensor size 1/2.55", pixel size 1.4µm, dual pixel PDAF, OIS, remote activation enabled | Samsung Galaxy S9  SM-G960U  Samsung Electronics Co., Ltd., Suwon-Shi, South Korea |
| Inflatable blood pressure cuff and attachable wall sphygmomanometer | Clinical-grade blood pressure cuff, child’s size 9 (15-21 cm) |  |
| Vinyl gloves, medium | Powder-free, transparent, ambidextrous, non-sterile, 2mm thick, synthetic vinyl | Powder-free Vinyl Examination Gloves  UPC #189365002574, Item No. DYND70401L  Primacare Medical Supplies, Paterson, New Jersey, USA |
| Nitrile gloves, small | Powder-free, black, general purpose, ambidextrous, non-sterile | Black Nitrile Pro Gloves  SMP-75042  STRONG Manufacturers, Pineville, North Carolina, USA |
| Nitrile gloves, medium | Powder-free, black, general purpose, ambidextrous, non-sterile | Black Nitrile Pro Gloves  SMP-75043  STRONG Manufacturers, Pineville, North Carolina, USA |
| Nitrile gloves, large | Powder-free, black, general purpose, ambidextrous, non-sterile | Black Nitrile Pro Gloves  SMP-75044  STRONG Manufacturers, Pineville, North Carolina, USA |
| Latex condoms | Male sex condom composed of latex material | Trojan condom  Church & Dwight Company, Ewing Township, New Jersey, USA |
|  |  | LifeStyles condom  LifeStyles HealthCare Pte Ltd.  Ansell Limited (previously known as Pacific Dunlop Limited), Richmond, Australia |
| Non-latex condoms | Male sex condom composed of synthetic polyisoprene material | Skyn condom  LifeStyles HealthCare Pte Ltd.  Ansell Limited, Richmond, Australia |
|  |  | Durex condom  Durex USA, Parsippany, New Jersey, USA  Reckitt Benckiser, Slough, United Kingdom |
| **Software and related tools** | | |
| Image labeling software | Online software for labeling images and other datasets. This software has a free tier, and an accompany python api (labelbox[data]) | Labelbox  <https://labelbox.com/> (Used April 2022)  San Francisco, CA, USA |
| Data analysis software | Python programming language with common statistical and analysis packages including numpy, pandas, and scipy, statsmodels |  |
| Graphical plotting software | The plotly package of python, which allows the creation of interactive and static figures (plots) that can be saved in file formats including .png, eps, and .html. | Plotly, version 5.7.0  Plotly Technologies Inc  <https://plot.ly/> (Used April 2022) |
|  | The matplotlib package of python, which allows the creation of static figures that can be salved in file formats including .jpg | matplotlib  The Matplotlib development team  <https://matplotlib.org/> (Used April 2022) |
| Version control system | All software was hosted on GitHub, an online version control system based on git | GitHub  Microsoft Corporation  San Francisco, CA, USA  <https://github.com/> |
| Tool to run code | All code was run using Google colab, a free cloud service tool to run code in python and other languages | Google Colaboratory  <https://colab.research.google.com/> |
