# Supplementary material for: Improving visualization of the cervix during pelvic exams: A simulation using a physical model of the speculum and human vagina as a steppingstone to reducing disparities in gynecological cancers
Source: PLoS One. 2023 Sep 26;18(9):e0283145. doi: 10.1371/journal.pone.0283145 (PMC10522035; doi:10.1371/journal.pone.0283145)
Supplement: S2 Table — (DOCX) [file pone.0283145.s003.docx]

S2 Table. Test-retest reliability and correlation comparing the measurements between the three trials of each condition combination.

|  | N ^a^ | Heise test-retest reliability^b^ | Pearson correlation coefficients | | | |
| --- | --- | --- | --- | --- | --- | --- |
|  |  |  | R₁₂ | R₁₃ | R₂₃ | mean R |
| **Height of Speculum Opening** | 11x3 | 0.970158 | 0.979470 | 0.993943 | 0.984493 | 0.985969 |
| **Relative Width (all)** | 50x3 | 0.887671 | 0.924046 | 0.914501 | 0.878502 | 0.905683 |
| *Relative Width at 40 mmHg* | 10x3 | 0.863271 | 0.846103 | 0.780755 | 0.796597 | 0.807819 |
| *Relative Width at 80 mmHg* | 10x3 | 0.911893 | 0.908187 | 0.940169 | 0.944005 | 0.930787 |
| *Relative Width at 120 mmHg* | 10x3 | 0.868165 | 0.910171 | 0.954343 | 0.910298 | 0.924937 |
| *Relative Width at 160 mmHg* | 10x3 | 0.837927 | 0.915694 | 0.981605 | 0.898240 | 0.931847 |
| *Relative Width at 200 mmHg* | 10x3 | 0.878994 | 0.936233 | 0.994459 | 0.933660 | 0.954784 |

Key values are highlighted in yellow. **R**, Pearson correlation coefficient; **R_12_**, correlation between measurements from 1^st^ and 2^nd^ trial; **R_13_**, correlation between measurements from 1^st^ and 3^rd^ trials s; **R_23_**, correlation between measurements from 2^nd^ and 3^rd^ trials; **mean R**, mean of **R_12_**, **R_13_**, and **R_23_**,

^a^ N representing the sample size is given as two numbers: number of sets x trials per set. Three trials were performed for every set.

^b^ Heise test-retest probability [43];
